# Isolated Hyperreligiosity in a Patient with Temporal Lobe Epilepsy

**DOI:** 10.1155/2015/235856

**Published:** 2015-08-13

**Authors:** Rocio Garcia-Santibanez, Harini Sarva

**Affiliations:** ^1^Department of Neurology, Mount Sinai Beth Israel, New York, NY 10003, USA; ^2^Department of Neurology, Maimonedes Medical Center, Brooklyn, NY 11219, USA

## Abstract

A 40-year-old man with history of temporal lobe epilepsy presented to the emergency department with hyperreligiosity after medication noncompliance. After medications were resumed, he returned to baseline. Many famous prophets are believed to have suffered epilepsy. Waxman and Geschwind described a group of traits in patients with temporal lobe epilepsy consisting of hyperreligiosity, hypergraphia, altered sexual behavior, aggressiveness, preoccupation with details, and circumstantiality. The incidence of religious experiences ranges from 0.3 to 3.1 percent in patients with epilepsy. Religious experiences can be ictal, interictal, or postictal. Treatment is aimed at the underlying seizure etiology.

## 1. Introduction

Epilepsy was associated with behavioral changes and religious fervor since the time of Hippocrates. Since these ancient times, seizures were thought to be due to demonic or divine influences. In fact, some believed that the visions and deep spiritual experiences of several famous prophets, including Ezekiel, Buddha, Mohammed, and Joan of Arc, were in fact epileptic in origin [1].

From the initial descriptions of personality changes, Gowers, Gibbs, and later Gastaut all described cognitive, memory, behavioral, and psychiatric changes in patients with epilepsy, in particular temporal lobe epilepsy (TLE) [2]. In 1974 Waxman and Geschwind described a constellation of personality traits in epileptics primarily with temporal lobe epilepsy [2]. The incidence of religious experiences ranges from 0.4 to 3.1 percent in patients with partial epilepsy [1].

We herein describe another case of this very rare condition presenting with only hyperreligiosity in a patient with TLE.

## 2. Case Report

A 40-year-old man, with a past medical history of temporal lobe epilepsy, presented to the emergency department with altered mental status for three days. As per his records, he was taking topiramate 100 mg twice daily and lamotrigine 200 mg twice daily, but compliance had been an issue.

On examination, he was uncooperative. He was constantly making religious remarks, saying “God is with me and I do not need doctors or medications.” He would interpret every question asked to him as questioning his faith and at times attempted to convert doctors and staff to Islam. He believed everyone around him was preventing him from obtaining salvation. The remainder of the neurological exam, including cranial nerves, motor examination, sensations, reflexes, coordination, and gait, was grossly normal. Initially, he was thought to have an acute psychotic episode. Psychiatry, however, was concerned that his religious comments and paranoia regarding his treatment in the hospital may be a part of his epilepsy.

His family was eventually contacted after multiple attempts and they reported that he had a generalized tonic-clonic seizure two days prior to being hospitalized and after the generalized seizure he started having increasing religious thoughts. The wife called an ambulance because she was frightened by his excessive religious speech. She was not sure when he stopped taking his medications. She reported that he had a history of epilepsy of unknown etiology since the age of 20. The most common seizure semiology was generalized tonic-clonic seizures but occasionally, he would have episodes of hyperreligiosity with secondarily generalized events. There was no mention of aura preceding any of the generalized seizures. These similar episodes of hyperreligiosity in the past occurred when he did not take his antiepileptic medications (AEDs). He did not have a history of psychiatric disease. An EEG showed right-sided frontotemporal sharp waves. His MRI was unremarkable which included thin cuts over temporal lobes and hippocampus. His hyperreligiosity was deemed to be due to his epileptic activity. He refused intravenous AEDs, as he believed it was antireligious poison but was convinced to take his home medications. After three days of treatment, he became more alert. His wife noted that he returned to baseline and was discharged home with follow-up with his neurologist.

## 3. Discussion

This case demonstrates the various psychiatric, behavioral, and cognitive changes, associated with temporal lobe epilepsy. His symptoms can be either ictal or postictal hyperreligiosity as described by Geshwind or epileptic psychosis [1–3]. Both presentations have a higher prevalence in temporal lobe epilepsy [1, 3].

Hyperreligiosity in epilepsy has been known to be part of the Gastaut-Geshwind syndrome which also consists of hypergraphia or excessive compulsive writing, altered sexual behavior, aggression, stickiness or viscosity, preoccupation with details, seriousness, and circumstantiality [2].

Hyperreligiosity may be an ictal, an interictal, or a postictal phenomenology. Ictal religiosity is a type of ecstatic seizure, such as feelings of joy or pleasure. Different examples of ictal religious experiences include intense emotions of God's presence, hallucinations of God's voice, clairvoyance, or even telepathy [1]. A religious aura for hours or days preceding ictal events in epileptics was also described [1]. Postictal hyperreligiosity is usually manifested as prolonged intense religiosity lasting hours to days; multiple reports have been published in which subjects had religious conversions after having a seizure [1]. Interictal religiosity presents as a heightened state of religious conviction with personality changes as described later in this discussion [1].

A handful of large studies provide better characterization of these events. In 1989, Roberts and Guberman showed in a study of 50 patients with temporal lobe epilepsy that 51 percent of these patients had an experience of salvation [4]. In a study of 234 patients [5], 1.3 percent had ictal religious experiences, usually associated with right temporal lobe origin. Experiences included a sense of presence of God and auditory or visual hallucinations of God [5]. A more recent study in 2014 showed that self-reported spirituality scores were higher not only in patients with temporal lobe epilepsy but also in epileptics with lower education level which may influence their response to these epileptic spiritual events [6]. Religious manifestations not only present with behavioral changes but also with motor manifestations. Lin et al. described the Sign of the Cross as an ictal hand automatism in four out of 530 patients [7]. All four patients had temporal lobe epilepsy. This report was done in Brazil, the country with the largest Catholic population in the world, possibly suggesting that the religious background might have played a role [7].

The pathophysiology of hyperreligiosity is complex and not fully understood. Religiosity in epilepsy is thought to originate in the limbic system due to its association with the temporal lobe and its function in emotions. Kindling, which refers to an enhancement of seizure susceptibility due to repeated stimulation [8], might be one of the ways hyperreligiosity develops as it is believed activation and strengthening of limbic-cortical connections occur in these patients [9]. Imaging studies have not completely elucidated the underlying changes. Wuerfel evaluated hippocampal MRI volumes in patients with refractory epilepsy and found a smaller right hippocampus in patients with hyperreligiosity but not with other components of the Gastaut-Geschwind syndrome, suggesting that the right hippocampus may have a role in the development of religiosity but causality could not be determined [10]. Involvement of the Papez circuit in the limbic system may lead to ictal events and ictal religiosity is more common with a right temporal seizure focus [1]. Chronic stimulation of the amygdala due to seizure activity may lead to altered behavior and heightened emotionality during the interictal period. This behavior is what is known as epileptic personality or interictal behavior. Geshwind described this as the interictal behavioral syndrome, which he observed in subjects with temporal lobe epilepsy who had a chronic change in personality which became more striking as time passes [2]. Postictal religiosity may be associated with bilateral temporal lobe dysfunction or a bilateral expression of a postictal state due to single focus [11]. This bilateral association was also described in postictal psychosis [1, 11].

Hyperreligiosity and psychosis are less common behavioral manifestations in epilepsy. Other more common behavioral changes include depression, anxiety, agitation, aggression, hyperactivity, inattention, attention deficit hyperactive disorder, and mood liability; up to 75% of patients with epilepsy have some behavioral comorbidities [12].

Our patient's symptoms may be difficult to categorize. The EEG changes and resolution with appropriate AED therapy suggest an ictal event. Although one could also argue it could have been a prolonged postictal event, we do not know what his baseline interictal pattern was and if his prior hyperreligious episodes resolved spontaneously. Epileptic psychosis is another possibility which we cannot ignore. As he was in the hospital, he was ensured to receive his appropriate medications.

Treatment of behavioral manifestations of epilepsy is aimed at the underlying epilepsy with antiepileptic medications. Prognosis depends on how quickly this semiology is recognized and treated [1]. Symptomatic management with psychiatric medications might be needed if the patient is at risk of injury [12].

In summary, hyperreligiosity is a known epileptic manifestation that may be an ictal, interictal, or postictal phenomenon, requiring appropriate diagnosis and treatment to prevent further neurologic injury.
